# First-trimester fatty liver index and hepatic steatosis index independently predict gestational diabetes risk: a prospective cohort study

**DOI:** 10.3389/fnut.2026.1769688

**Published:** 2026-03-19

**Authors:** Rong Shuai, Lizhong Lin, Dongqian Yang, Yuqin Shen, Li Zhang

**Affiliations:** 1 Department of Laboratory Medicine, Changde Hospital, Xiangya School of Medicine, Central South University (The First People's Hospital of Changde City), Changde, China; 2 Department of Obstetrics, Changde Hospital, Xiangya School of Medicine, Central South University (The First People's Hospital of Changde City), Changde, China

**Keywords:** fatty liver index, gestational diabetes mellitus, hepatic steatosis index, logistic regression model, predictors, prospective cohort study, ROC

## Abstract

**Background:**

Gestational diabetes mellitus (GDM) is a prevalent pregnancy complication characterized by complex metabolic pathophysiology. Although both the fatty liver index (FLI) and hepatic steatosis index (HSI) are well-established non-invasive markers for hepatic steatosis, their predictive value for GDM risk in the Korean population remains underexplored.

**Methods:**

This prospective cohort study enrolled 573 singleton pregnancies. FLI and HSI were calculated at 10–14 weeks of gestation. GDM was diagnosed via a two-step oral glucose tolerance test (OGTT) at 24–28 weeks of gestation. Multivariable logistic regression models were applied to assess the associations between FLI/HSI and GDM risk. Sensitivity and subgroup analyses were performed to verify the robustness of the findings, while receiver operating characteristic (ROC) curve analyses were conducted to evaluate the predictive performance of the two indices.

**Results:**

The mean age of the participants was 32.1 years, with a mean FLI of 18.0 and a mean HSI of 30.0. The overall incidence of GDM was 6.3%. After adjusting for confounding factors (age, high-density lipoprotein cholesterol (HDL-C), homeostasis model assessment of insulin resistance (HOMA-IR), free fatty acid (FFA), and gestational hypertension or preeclampsia), each 1-unit increase in FLI was associated with a 5% higher risk of GDM [odds ratio (OR) = 1.05, 95% confidence interval (CI): 1.03–1.06]. Similarly, each 1-unit increase in HSI correlated with a 19% elevated GDM risk (OR = 1.19, 95% CI: 1.11–1.28). Sensitivity and subgroup analyses further confirmed the stability of these associations. FLI exhibited superior predictive ability for GDM compared with HSI, with an area under the curve (AUC) of 0.8133 (95% CI: 0.7275–0.8991) versus 0.7868 (0.7075–0.8661) for HSI.

**Conclusion:**

Both FLI and HSI independently predict GDM risk in Korean women, with FLI demonstrating marginally better performance. Their early-pregnancy assessment (10–14 weeks) may enhance risk stratification and enable targeted preventive strategies.

## 1 Introduction

Gestational diabetes mellitus (GDM), a prevalent metabolic disorder during pregnancy, affects 15% of pregnant women globally (1). Notably, recent epidemiological studies have reported that the prevalence of GDM in Korean women ranges from 12.7% (2), which is slightly lower than the global average but still represents a significant public health burden—highlighting the need for targeted early screening strategies in this specific population. It not only adversely impacts maternal health but also increases long-term risks of type 2 diabetes (3, 4) and cardiovascular diseases in offspring (5–7).

Clinically, GDM is diagnosed via oral glucose tolerance tests (OGTT) at 24–28 weeks of gestation (8), but this window has notable limitations: irreversible fetal/maternal metabolic alterations and established *β*-cell dysfunction may already occur by this stage (9), highlighting the urgency of early prediction. Timely identification of high-risk women not only improves maternal and neonatal outcomes (10, 11) but also enables effective preventive interventions (12, 13), while accurate prediction models established before 18 weeks of gestation can provide critical scientific support for early management (14, 15).

The liver plays a pivotal role in maintaining blood glucose homeostasis. Hepatic lipid accumulation can induce insulin resistance by disrupting lipid metabolism, subsequently triggering abnormalities in glucose metabolism (16–20). The hepatic steatosis index (HSI) (21), a well-validated non-invasive biomarker, effectively quantifies the degree of hepatic fat accumulation. Studies have shown that there is a significant correlation between this condition and the risk of diabetes in the general population (22). Concurrently, another widely used non-invasive indicator—the fatty liver index (FLI)—integrates additional metabolic parameters, including body mass index (BMI), waist circumference, triglycerides (TG), and gamma-glutamyltransferase (GGT) (23, 24). Studies indicate FLI also exhibits a positive correlation with the risk of developing type 2 diabetes mellitus (T2DM) (25). However, the specific association between these two indicators and the risk of GDM remains poorly elucidated, with particularly scarce evidence in specific ethnic cohorts.

In recent years, the prevalence of non-alcoholic fatty liver disease (NAFLD)—a core driver of hepatic steatosis—has shown a sharp upward trend among Korean women (26). Furthermore, Korea’s healthcare system routinely incorporates comprehensive assessments of NAFLD-related biomarkers such as HSI and FLI into prenatal checkups, providing a robust foundation for epidemiological research. It should be noted that NAFLD mentioned in this study has been recently redefined as metabolic dysfunction-associated steatotic liver disease (MASLD) (27) via international consensus, referring to metabolic dysfunction-associated hepatic steatosis and related liver diseases (without the need to exclude alcohol intake), with hepatic fat accumulation as the core diagnostic feature.

This study is a secondary analysis of prospective cohort data from an original investigation examining the relationship between NAFLD and pregnancy outcomes (28). It collected comprehensive baseline data required for calculating the FLI and HSI during early pregnancy (10–14 weeks), including liver enzymes, metabolic parameters, and anthropometric measurements. GDM was diagnosed at 24–28 weeks, and sufficient covariates were available to control for confounding factors. The aim of this study was to assess the independent association between FLI and HSI during early pregnancy, thereby providing a scientific basis for the early risk stratification of GDM.

## 2 Methods

### 2.1 Data source

The study utilized de-identified data from a previously published open-access cohort study by Lee et al. investigating NAFLD and pregnancy outcomes (28). All analyses were conducted in compliance with terms of the Creative Commons Attribution 4.0 International (CC BY) license, including proper attribution to the original authors.

### 2.2 Study population

This secondary analysis utilized data from a prospective cohort study (ClinicalTrials.gov NCT02276144) conducted at two Korean tertiary centers between November 2014 and July 2016 (28). The original study enrolled 623 singleton pregnant women ≤14 gestational weeks after excluding those with pre-gestational diabetes, chronic liver disease, or excessive alcohol consumption. Ethical approvals were obtained from Seoul National University Boramae Medical Center and the Korean Ministry of Health and Welfare Institutional Review Boards, with all participants providing written informed consent by the Declaration of Helsinki.

**Figure 1:**
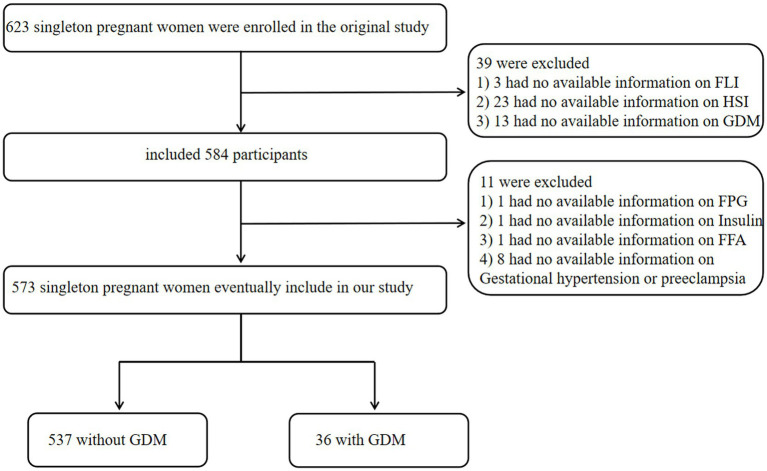
Flowchart of study participants.

Following rigorous exclusion criteria, we sequentially excluded: (1) 23 participants lacking essential HSI components; (2) 3 participants lacking essential FLI components (3) 13 with undetermined GDM status; (4) 11 participants with incomplete covariate variables data (1 missing FPG (fasting plasma glucose), 1 missing insulin, and 9 lacking gestational hypertension/preeclampsia records). After these exclusions, the final analytic cohort comprised 573 women, including 36 GDM cases and 537 non-GDM controls. This selection process ensured complete data availability for all key exposure (FLI and HSI), outcome (GDM), and covariate variables (Figure 1).

### 2.3 Variables

#### 2.3.1 Xposure variable

Using FLI as the exposure variable, the formula included BMI, waist circumference, TG, and GGT. According to FLI, the patients were divided into three groups: low-risk group (FLI ≤ 20), intermediate risk group (20 < FLI < 60), and high risk group (FL I ≥ 60) (29).


$$\mathrm{FLI}=\frac{\exp\;[\text{Mode}l_{\mathrm{FLI}}]\;}{1+\exp\;[\text{Mode}l_{\mathrm{FLI}}]}\times 100$$

where $\begin{array}{l} \text{Mode}l_{\mathrm{FLI}}=0.953\times \ln\;[\mathrm{TG}\;(\mathrm{mg}/\mathrm{dL})]+0.139\times \mathrm{BMI}\;(\mathrm{kg}/m^{2})\\ +0.718\times \ln\;[\mathrm{GGT}\;(\mathrm{IU}/L)]+(0.053\times \text{waist circumference}[\mathrm{cm}])\\ -15.745 \end{array}$

The HSI was the exposure variable, and the formula included ALT, being alanine aminotransferase, and AST, being aspartate aminotransferase (30). Participants were stratified into three groups according to HSI: low risk of steatosis (HSI < 30), intermediate risk (30 ≤ HSI ≤ 36), and high risk (HSI > 36).


$$\mathrm{HSI}=8\times (\mathrm{ALT}\;[\mathrm{IU}/L]/\mathrm{AST}\;[\mathrm{IU}/L])+\mathrm{BMI}\;(\mathrm{kg}/m^{2})+2$$

#### 2.3.2 Outcome variable

GDM was diagnosed using the two-step approach recommended as the standard diagnostic protocol by the American College of Obstetricians and Gynecologists (ACOG) at 24–28 weeks of gestation (31), in accordance with the methodology implemented in the original cohort study (28). First, all participants underwent a non-fasting 50 g oral glucose challenge test. A 1-h post-load glucose level ≥7.8 mmol/L was considered a positive screening result. After an overnight fast, those who tested positive proceeded to a diagnostic 100 g OGTT. GDM was confirmed if two or more glucose values met or exceeded the following thresholds: fasting glucose ≥5.3 mmol/L, 1-h glucose ≥10.0 mmol/L, 2-h glucose ≥8.6 mmol/L.

#### 2.3.3 Covariates

Covariates for the multivariable logistic regression model were selected based on clinical relevance and statistical criteria. First, stepwise logistic regression was used to screen for potential confounding factors, with variables retained if they altered the regression coefficients of exposure factors by ≥10%. Subsequently, multicollinearity was assessed via the Variance Inflation Factor (VIF), and variables with a VIF ≥ 5 were excluded. The final model included age, high-density lipoprotein cholesterol (HDL-C), homeostasis model assessment of insulin resistance (32) (HOMA-IR, calculated as [FPG (mmol/L) × insulin (μIU/mL)]/22.5), free fatty acid (FFA), and gestational hypertension or preeclampsia. These covariates belonged to distinct metabolic domains and had no significant intercorrelation.

### 2.4 Statistical analysis

Missing data were addressed using complete case analysis. Participants with missing values in exposure variables (FLI, HSI, or their components), outcome variables (GDM), or any of the selected covariates (age, HDL-C, HOMA-IR, FFA, and gestational hypertension or preeclampsia) were excluded from the analytical dataset. This approach was adopted to ensure a uniform analytic sample across all regression models and to avoid potential bias introduced by imputation methods, given that the proportion of missing data was small.

Continuous variables were presented as mean±standard deviation (normal distribution) or median with interquartile range (skewed distribution), while categorical variables were expressed as frequencies (percentages). Group comparisons were performed using one-way ANOVA (normal distribution), Kruskal-Wallis test (skewed distribution), or chi-square test (categorical variables). Logistic regression models were constructed to evaluate the association between HSI and GDM: Model 1 (unadjusted), Model 2 (adjusted for age), and Model 3 (fully adjusted for age, HDL-C, HOMA-IR, FFA, and Gestational hypertension or preeclampsia). Generalized additive models (GAM) were employed to examine potential non-linear relationships. In the GAM analysis, the exposure variables (FLI and HSI) were modeled as smooth terms using cubic regression splines, with smoothing parameters selected automatically via the generalized cross-validation criterion. The models were specified with a binomial family and logit link function. All adjusted covariates were included as linear terms in the models. Sensitivity analyses included: (1) excluding participants with gestational hypertension or preeclampsia, (2) excluding participants with HOMA-IR ≥ 2, (3) trend tests through FLI and HSI were divided into low, intermediate, and high risk. Subgroup analyses stratified by age (35/≥35 years), parity, pre-pregnancy BMI (<25/≥25 kg/m^2^), HOMA-IR (≤2/>2), GGT (20/≥20 IU/L), and NAFLD status were conducted with interaction tests using likelihood ratio tests. Receiver operating characteristic (ROC) analysis determined the predictive performance. All analyses were performed using R software (version 4.2.2) with two-tailed *p* < 0.05; statistically significant results were considered following STROBE guidelines.

## 3 Results

### 3.1 Baseline characteristics

The baseline characteristics of the 573 study participants, stratified by GDM status, are presented in Table 1. No significant differences were observed in age or parity between groups. Compared with non-GDM individuals, participants with GDM exhibited pronounced metabolic disturbances, including higher BMI, elevated liver enzymes (ALT, GGT), adverse lipid profiles (higher TG, lower HDL-C), and pronounced insulin resistance (higher FPG, insulin levels, and proportion with HOMA-IR ≥ 2). Ultrasound findings revealed a significantly higher prevalence of NAFLD in the GDM group than in the non-GDM group (55.6% vs. 16.8%, *p* < 0.001). Additionally, there were significant differences in both the FLI and HSI between the GDM and non-GDM groups (both *p* < 0.001): the median FLI in the GDM group [39.8 (24.5, 68.7)] was significantly higher than that in the non-GDM group [10.9 (6.0, 20.3)], the mean HSI (35.7 ± 5.8) was significantly higher than that in the non-GDM group (30.0 ± 4.5).

**Table 1:** The baseline characteristics of study participants.

Variables	Total (n = 573)	Non-GDM(n = 537)	GDM (n = 36)	*p*-value
Age (years)	32.1 ± 3.8	32.0 ± 3.8	32.6 ± 3.6	0.408
Parity				0.99
No	303 (52.9)	284 (52.9)	19 (52.8)	
Yes	270 (47.1)	253 (47.1)	17 (47.2)	
BMI (kg/m^2^)	22.1 ± 3.5	21.8 ± 3.2	25.8 ± 5.2	< 0.001
AST (IU/L)	16.0 (14.0, 20.0)	16.0 (14.0, 20.0)	17.5 (15.0, 21.2)	0.066
ALT (IU/L)	11.0 (8.0, 15.0)	11.0 (8.0, 14.0)	15.0 (10.0, 25.5)	0.003
GGT (IU/L)	12.0 (10.0, 15.0)	12.0 (10.0, 15.0)	15.5 (11.0, 23.0)	0.002
TC (mg/dL)	170.0 (154.0, 190.0)	169.0 (153.0, 190.0)	179.5 (156.8, 196.8)	0.14
TG (mg/dL)	110.0 (87.0, 141.0)	107.0 (85.0, 136.0)	159.0 (128.5, 194.2)	< 0.001
HDL-C (mg/dL)	64.8 (55.0, 73.4)	65.0 (55.6, 73.8)	60.5 (46.8, 71.0)	0.044
LDL-C (mg/dL)	83.6 (69.6, 97.9)	83.6 (69.6, 97.7)	82.6 (71.7, 98.2)	0.9
FPG (mg/dL)	76.9 ± 9.7	76.4 ± 9.0	84.6 ± 15.2	< 0.001
Insulin (μIU/mL)	8.4 (5.3, 11.5)	8.0 (5.3, 11.1)	15.3 (8.9, 21.9)	< 0.001
HOMA-IR				< 0.001
<2	382 (66.7)	370 (68.9)	12 (33.3)	
≥2	191 (33.3)	167 (31.1)	24 (66.7)	
Gestational hypertension or preeclampsia				0.014
No	557 (97.2)	525 (97.8)	32 (88.9)	
Yes	16 (2.8)	12 (2.2)	4 (11.1)	
NAFLD, *n* (%)				< 0.001
No	463 (80.8)	447 (83.2)	16 (44.4)	
Yes	110 (19.2)	90 (16.8)	20 (55.6)	
FFA (μEq/L)	609.0 (459.0, 786.0)	601.0 (458.0, 778.0)	737.5 (470.5, 924.2)	0.034
FLI	11.5 (6.1, 22.8)	10.9 (6.0, 20.3)	39.8 (24.5, 68.7)	< 0.001
FLI, *n* (%)				< 0.001
≤20 (low risk)	406 (70.9)	399 (74.3)	7 (19.4)	
21–59 (intermediate risk)	137 (23.9)	120 (22.3)	17 (47.2)	
≥60 (high risk)	30 (5.2)	18 (3.4)	12 (33.3)	
HSI	30.4 ± 4.8	30.0 ± 4.5	35.7 ± 5.8	< 0.001
HSI, *n* (%)				< 0.001
<30 (low risk)	316 (55.1)	310 (57.7)	6 (16.7)	
30–36 (intermediate risk)	188 (32.8)	172 (32)	16 (44.4)	
>36 (high risk)	69 (12.0)	55 (10.2)	14 (38.9)	

GDM, gestational diabetes mellitus; parity (No: Never given birth; Yes: Have given birth); BMI: body mass index; AST, aspartate aminotransferase; ALT, alanine aminotransferase; GGT, gamma-glutamyl transferase; TC, total cholesterol; TG, triglyceride; HDL-C, high-density lipoprotein cholesterol; LDL-C, low-density lipid cholesterol; FPG, fasting plasma glucose; HOMA-IR, homeostasis model assessment-insulin resistance; NAFLD, nonalcoholic fatty liver disease; FFA, Free fatty acid; FLI, Fatty liver index; HSI, hepatic steatosis index.

### 3.2 Univariate logistic regression analysis of factors influencing GDM

Univariate logistic regression analysis (Table 2) identified multiple significant predictors of GDM. Metabolic parameters (BMI, ALT, GGT, TG, FPG, insulin, HOMA-IR), clinical conditions (gestational hypertension/preeclampsia, NAFLD), and liver steatosis indices (FLI, HSI) all demonstrated strong positive associations with GDM (all *p* < 0.05). HDL-C exhibited a protective effect (*p* = 0.007), while age, parity, AST, TC, LDL-C, and FFA showed no significant associations (all *p* > 0.05).

**Table 2:** Univariate logistic regression models evaluating the association of demographic, biochemical, and clinical characteristics with GDM.

Variable	OR-95CI	*P-*value
Age	1.04 (0.95–1.14)	0.408
Parity		
No	Ref	
Yes	1 (0.51–1.97)	0.99
BMI (kg/m^2^)	1.27 (1.17–1.38)	<0.001
AST (IU/L)	1.02 (0.99–1.05)	0.189
ALT (IU/L)	1.04 (1.01–1.06)	0.002
GGT (IU/L)	1.03 (1.01–1.06)	0.014
TC (mg/dL)	1.01 (1–1.02)	0.088
TG (mg/dL)	1.02 (1.01–1.02)	<0.001
HDL-C (mg/dL)	0.96 (0.94–0.99)	0.007
LDL-C (mg/dL)	1 (0.98–1.02)	0.996
FPG (mg/dL)	1.07 (1.04–1.11)	<0.001
Insulin (μIU/mL)	1.12 (1.07–1.17)	<0.001
HOMA-IR	1.47 (1.22–1.78)	<0.001
Gestational hypertension or preeclampsia		
No	Ref	
Yes	5.47 (1.67–17.91)	0.005
NAFLD		
No	Ref	
Yes	6.21 (3.1–12.44)	<0.001
FFA (μEq/L)	1.00 (1.00–1.01)	0.032
FLI(per 1-unit)	1.05 (1.04–1.07)	<0.001
FLI (per 1-SD)	2.55 (1.98–3.28)	<0.001
HSI(per 1-unit)	1.22 (1.15–1.3)	<0.001
HSI (per1-SD)	2.62 (1.93–3.55)	<0.001

OR, odds ratio; CI, confidence interval.

### 3.3 The associations of FLI and HSI with GDM

**Figure 2:**
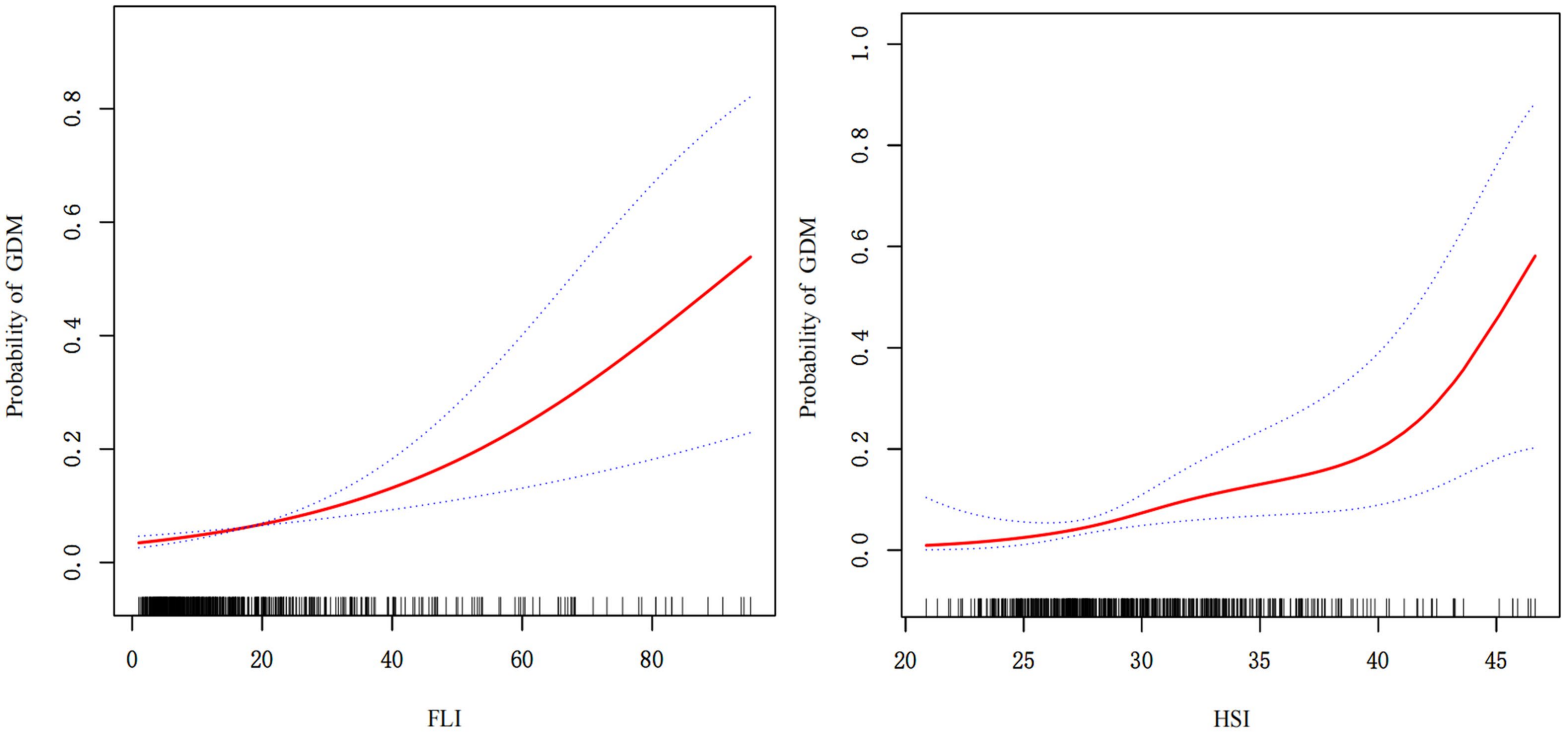
Association of FLI and HSI with GDM. FLI and HSI were linearly associated with GDM in GAM. The solid radial line represents a smooth curve fit between variables. The blue band indicates the 95% confidence interval of the fit. Adjusted for age, HDL-C, HOMA-IR, FFA, and gestational hypertension or preeclampsia.

Table 3 demonstrates significant graded associations between hepatic steatosis indices and GDM risk across three multivariate models. After full adjustment for covariates (age, HDL-C, HOMA-IR, FFA, and gestational hypertension or preeclampsia), each unit increase in FLI was associated with a 5% higher GDM risk, and high-risk FLI conferred a 22.38-fold increased risk compared with low-risk FLI. For HSI, each unit increase correlated with a 19% elevated GDM risk. Compared with low-risk HSI, and high-risk HSI conferred an 8.31-fold higher risk. Generalized additive models confirmed a linear dose–response relationship between both FLI/HSI and GDM risk (Figure 2).

**Table 3:** Multivariate logistic regression models evaluating the associations of hepatic steatosis with GDM.

Variable	Model 1		Model 2		Model 3	
	OR (95%CI)	*p* value	OR (95%CI)	*P* value	OR (95%CI)	*P* value
FLI (per 1-unit)	1.05 (1.04–1.07)	<0.001	1.05 (1.04–1.07)	<0.001	1.05 (1.03–1.06)	<0.001
FLI (per 1-SD)	2.55 (1.98–3.28)	<0.001	2.54 (1.98–3.27)	<0.001	2.25 (1.71–2.97)	<0.001
FLI						
≤20 (low risk)	1(Ref)		1(Ref)		1(Ref)	
21–59 (intermediate risk)	8.07 (3.27–19.93)	<0.001	7.97 (3.22–19.7)	<0.001	6.79 (2.63–17.5)	<0.001
≥60 (high risk)	38 (13.37–108.04)	<0.001	37.85 (13.3–107.68)	<0.001	22.38 (7.14–70.19)	<0.001
*P* for trend		<0.001		<0.001		<0.001
HSI (per 1-unit)	1.22 (1.15–1.3)	<0.001	1.23 (1.15–1.31)	<0.001	1.19 (1.11–1.28)	<0.001
HSI (per 1-SD)	2.62 (1.93–3.55)	<0.001	2.66 (1.95–3.62)	<0.001	2.31 (1.65–3.22)	<0.001
HSI						
<30 (low risk)	1 (Ref)		1 (Ref)		1 (Ref)	
30–36 (intermediate risk)	4.81 (1.85–12.51)	0.001	4.83 (1.85–12.58)	0.001	4.56 (1.69–12.3)	0.003
>36 (high risk)	13.15 (4.85–35.69)	<0.001	13.17 (4.85–35.79)	<0.001	8.31 (2.83–24.39)	<0.001
*P* for trend		<0.001		<0.001		<0.001

Model 1 was adjusted for nothing.

Model 2 was adjusted for the variable age.

Model 3 was adjusted for all covariates in model 2 + HDL-C, HOMA-IR, FFA, and Gestational hypertension or preeclampsia.

### 3.4 Sensitivity analyses

Sensitivity analyses (Table 4) confirmed the robustness of the associations. After excluding participants with gestational hypertension/preeclampsia (Model I) or HOMA-IR ≥ 2 (Model II), FLI and HSI remained significantly associated with GDM risk, though the associations were attenuated in Model II. Trend tests across FLI/HSI risk stratifications further supported the reliability of the findings.

**Table 4:** Relationship between hepatic steatosis and GDM risk in different sensitivity analyses.

Variable	Model I		Model II	
	OR (95%CI)	*P* value	OR (95%CI)	*P* value
FLI (per 1-unit)	1.05 (1.03–1.06)	<0.001	1.05 (1.02–1.07)	<0.001
FLI (per 1-SD)	2.34 (1.75–3.13)	<0.001	2.26 (1.45–3.51)	<0.001
FLI				
≤20 (low risk)	1 (Ref)		1 (Ref)	
21–59 (intermediate risk)	8.02 (2.95–21.81)	<0.001	4.78 (1.3–17.54)	0.018
≥60 (high risk)	24.68 (7.33–83.13)	<0.001	31.19 (4.38–222.15)	0.001
*P* for trend		<0.001		<0.001
HSI (per 1-unit)	1.2 (1.11–1.29)	<0.001	1.24 (1.1 ~ 1.4)	0.001
HSI (per 1-SD)	2.36 (1.67–3.33)	<0.001	2.76 (1.55–4.9)	0.001
HSI				
<30 (low risk)	1 (Ref)		1 (Ref)	
30–36 (intermediate risk)	5.29 (1.82–15.36)	0.002	3.51 (0.85–14.57)	0.083
>36 (high risk)	10.78 (3.44–33.79)	<0.001	11.51 (2.13–62.26)	0.005
*P* for trend		<0.001		0.004

Model I was a sensitivity analysis after excluding those with gestational hypertension or preeclampsia, *n* = 557. We adjusted for age, HDL-C, HOMA-IR, and FFA.

Model II was a sensitivity analysis after excluding those with HOMA-IR≥2, *n* = 382. We adjusted for age, HDL-C, FFA, and gestational hypertension or preeclampsia.

### 3.5 Subgroup analyses

**Figure 3:**
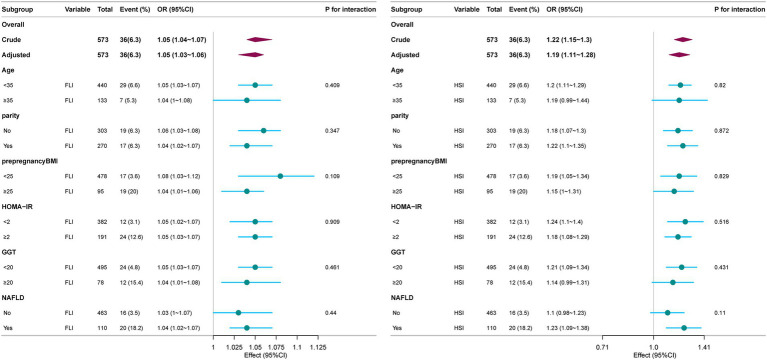
Forest plot of the effect size of FLI and HSI on GDM in subgroups. Each stratification was adjusted for age, HDL-C, HOMA-IR, FFA, and gestational hypertension or preeclampsia, except the stratification factor itself.

Subgroup analyses were performed to evaluate the associations between hepatic steatosis indices (FLI and HSI) and GDM risk across different populations (Figure 3). For FLI, the positive association persisted in subgroups stratified by age, parity, and HOMA-IR, with no significant interaction effects (*p* for interaction >0.05). Notably, the association was attenuated in participants with pre-pregnancy BMI ≥ 25 kg/m^2^ (OR = 1.03, 95% CI: 0.99–1.07) or GGT ≥ 20 IU/L (OR = 1.03, 95% CI: 0.99–1.07). For HSI, the association remained significant in most subgroups, including younger women (35 years), nulliparous women, and those with HOMA-IR < 2. However, the association was weaker in older women (≥35 years) and those with NAFLD.

### 3.6 ROC analyses

**Figure 4:**
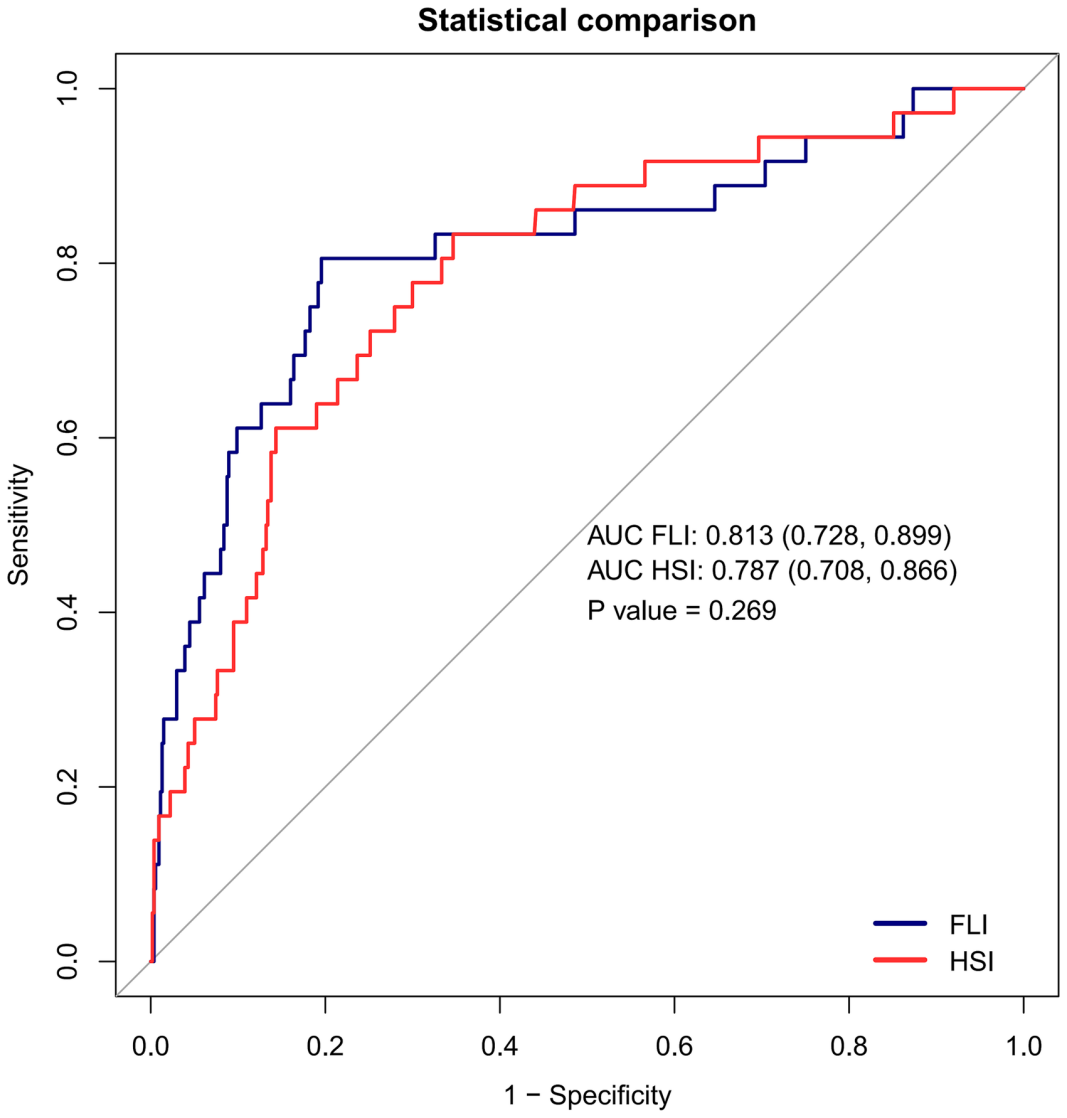
ROC curves for FLI, HSI, and GDM in pregnant women. The results show the AUC of the FLI was 0.8133, AUC of HSI was 0.7868. The AUC of FLI was slightly higher than that of HSI.

ROC analysis (Figure 4 and Table 5) showed that FLI had superior predictive performance for GDM compared with HSI (AUC = 0.8133, 95%CI:0.7275–0.8991 vs. AUC = 0.7868, 95%CI:0.7075–0.8661). The optimal cutoff value for FLI was 23.33 (sensitivity = 80.6%, specificity = 80.5%), and for HSI was 31.13 (sensitivity = 83.3%, specificity = 65.36%).

**Table 5:** Areas under the receiver operating characteristic curves (AUROC) for evaluating hepatic steatosis in identifying GDM.

Variable	AUC (95%CI)	Best threshold	Specificity	Sensitivity	Youden index
FLI	0.8133 (0.7275–0.8991)	23.33	0.8045	0.8056	0.6101
HSI	0.7868 (0.7075–0.8661)	31.13	0.6536	0.8333	0.4869

## 4 Discussion

This prospective cohort study of 573 Korean pregnant women demonstrated that both the FLI and HSI measured at 10–14 weeks of gestation independently predict GDM risk. Each 1-unit increase in FLI was associated with a 5% higher GDM risk (OR = 1.05), while each 1-unit increase in HSI correlated with a 19% elevated risk (OR = 1.19). FLI showed marginally superior predictive performance (AUC: 0.8133 vs. 0.7868 for HSI). Early-pregnancy assessment of these indices may improve GDM risk stratification and targeted prevention.

The superior predictive capacity of FLI can be attributed to its more comprehensive integration of metabolic and anthropometric parameters. Unlike HSI, which only incorporates ALT/AST ratio and BMI, FLI includes TG, WC, BMI, and GGT. TG is a key marker of lipid metabolism dysfunction, a core contributor to insulin resistance during pregnancy. WC reflects visceral adiposity, which induces systemic inflammation and exacerbates hepatic insulin resistance. GGT not only indicates liver fat accumulation but also reflects oxidative stress, a critical mediator of glucose metabolism disorders in pregnancy. By integrating these multiple metabolic dimensions, FLI captures a more holistic picture of the metabolic derangements that precede GDM, explaining its superior discriminatory ability compared to the more narrowly focused HSI.

Previous studies have reported associations between FLI/HSI and diabetes risk, but notable differences in effect sizes exist compared to the present findings. Zhu et al. (25) observed a 1.9% increase in T2DM risk per unit increase in FLI in a Japanese general population, which is lower than the 5% increase in GDM risk observed here. Cai et al. (33) reported that a 1-standard-deviation (SD) increase in HSI was associated with a 62% higher T2DM risk in Chinese adults, whereas our study found a 2.31-fold higher GDM risk per 1-SD HSI increase. These discrepancies may stem from several factors: first, pregnancy-induced physiological changes (e.g., increased insulin resistance and lipid accumulation) may amplify the impact of hepatic steatosis on glucose metabolism, making FLI/HSI more sensitive predictors of GDM than T2DM. Second, GDM and T2DM differ in pathophysiology—GDM is primarily driven by inadequate adaptation of *β*-cell function to pregnancy-related insulin resistance, whereas T2DM involves long-term pancreatic β-cell dysfunction and systemic metabolic decompensation. Third, the Korean pregnant population in our study may have distinct metabolic characteristics compared to general populations or other ethnic groups, further contributing to effect size variations. Linder et al. (34) demonstrated associations between early pregnancy FLI/HSI and GDM risk in 109 Austrian women. Our study further validated these independent associations in a larger cohort of 573 Korean pregnant women. Despite different ethnic backgrounds, the consistent findings across both studies suggest that these non-invasive indices are robust cross-ethnic predictors of GDM, supporting their broader applicability to diverse populations.

Notably, the FLI/HSI values in our study may reflect pregnancy-related metabolic changes rather than pre-pregnancy baseline liver fat. During early pregnancy, hormonal shifts (e.g., increased estrogen and progesterone) and altered energy metabolism lead to progressive insulin resistance and lipid accumulation. The FLI and HSI measured at 10–14 weeks likely capture these early pregnancy-specific metabolic adaptations, which are directly relevant to GDM development. This aligns with our finding that these indices are robust predictors of subsequent GDM, as they reflect the dynamic metabolic state of pregnancy rather than static pre-pregnancy liver fat levels.

For the subgroups of pre-pregnancy BMI ≥ 25 kg/m^2^and GGT ≥ 20 IU/L, the attenuated effect sizes observed may be explained by multiple pregnancy-specific metabolic and statistical factors. First, these high-risk subgroups had markedly higher event rates than the overall population (20.0% for BMI ≥ 25 kg/m^2^, 15.4% for GGT ≥ 20 IU/L vs. 6.3% overall), and their relatively small sample sizes (n = 95 for BMI ≥ 25 kg/m^2^, n = 78 for GGT ≥ 20 IU/L) may have led to insufficient statistical power and the contraction of effect estimates. Second, in individuals with elevated pre-pregnancy BMI, obesity itself acts as a robust independent predictor of gestational diabetes mellitus, potentially masking the independent associative effect of FLI on the outcome. Additionally, insulin resistance in obese individuals is likely driven primarily by peripheral adipose tissue dysfunction rather than hepatic steatosis per se, which may reduce the relative predictive importance of liver-based markers.

NAFLD is considered a potential risk factor for insulin resistance and may be closely related to the occurrence of GDM (35). Although liver biopsy is the gold standard for the diagnosis of NAFLD, it is not suitable for routine screening in asymptomatic pregnant women due to its invasive nature. In contrast, liver ultrasound, as a non-invasive examination method, has certain value in the early identification of NAFLD during pregnancy (36). Previous studies have found that NAFLD diagnosed by ultrasound in the first trimester can predict abnormal glucose or GDM in the second trimester (35). However, the low sensitivity of liver ultrasound for mild fatty liver may affect its diagnostic accuracy. In recent years, FLI and HSI, as simple and non-invasive screening tools for NAFLD, have shown good applicability in populations (37). This study supports a dose–response relationship between FLI/HSI and GDM risk. Even in pregnant women with normal liver ultrasound results, abnormal FLI is still associated with an increased risk of GDM, suggesting that this index may also have an important predictive value for GDM in pregnant women with mild to moderate fatty liver.

There is a correlation between lipid metabolism disorders and GDM, indicating the existence of metabolic pathways between them. The core mechanism of this condition is the disruption of the insulin signalling pathway, which is caused by ectopic lipid deposition. The abnormal accumulation of lipid metabolites in tissues such as the liver has been demonstrated to interfere with insulin receptor signalling, thereby inducing hepatic insulin resistance (38, 39). This pathological process assumes particular significance during pregnancy, as the physiological decrease in insulin sensitivity (40, 41) that occurs during this period serves to exacerbate hepatic gluconeogenesis further, ultimately resulting in significant hyperglycemia in women who exhibit higher FLI and HSI scores. It is important to note that such metabolic disturbances may persist into the postpartum period. Research has indicated that, in non-obese pregnant women with GDM, the lipid content of hepatocytes persists at an abnormally elevated level post-partum (42, 43). This finding suggests the potential for hepatic lipid metabolism disorder to be a distinctive feature of these patients. This abnormality is closely associated with insulin resistance, chronic low-grade inflammation, and a substantially increased risk of developing type 2 diabetes over the subsequent decade (44, 45). Collectively, these findings indicate that fatty liver functions as a dual risk factor for GDM, thereby serving as a pivotal conduit between metabolic abnormalities during pregnancy and the development of long-term diabetes.

This study provides several notable strengths. First, we established a novel independent association between first-trimester FLI and HSI levels and subsequent GDM risk, with robust adjustment for metabolic confounders. Second, the prospective design with standardized biomarker measurements at 10–14 weeks of gestation enhances temporal causality. Third, combined sensitivity analyses (excluding high-risk subgroups (gestational hypertension or HOMA-IR ≥ 2)) and subgroup analyses consistently supported the main results. Fourth, we determined clinically relevant FLI and HSI thresholds for GDM prediction through ROC analysis, demonstrating its potential as an early screening tool.

This study has several limitations that warrant consideration. First, as a secondary analysis of existing cohort data, we were limited by the variables originally collected. This prevented us from assessing important potential confounders such as family history of diabetes, smoking, diet, physical activity. Despite adjusting for key metabolic parameters, in our multivariate models, residual confounding by these unmeasured factors cannot be completely ruled out. Second, the potential for measurement bias inherent in secondary data analysis should be acknowledged. For instance, while the original study followed standardized protocols, subtle variations in the collection of anthropometric data (e.g., waist circumference) or the assay of biological samples (e.g., blood biomarkers) across centers could have introduced potential, non-differential measurement error into the calculation of FLI and HSI. Third, the single-centre design and exclusive focus on Korean women may also limit the generalisability of our findings to other ethnic populations with different metabolic characteristics. Finally, FLI and HSI were assessed at a single time point (10–14 weeks of gestation), and their dynamic changes throughout pregnancy and potential association with GDM risk were not evaluated (46). Future studies should be conducted in more diverse populations, incorporate comprehensive assessment of confounders, and implement dynamic monitoring of FLI and HSI during the second and third trimesters. Simultaneously, prospective cohort studies specifically designed to validate hepatic steatosis indices for GDM prediction are needed to address the issues of data integrity and measurement bias inherent in secondary analyses.

## 5 Conclusion

This study reveals that both FLI and HSI can independently predict GDM risk in Korean women, with FLI performing slightly better than HSI. Evaluating these two indicators in the first trimester (weeks 10–14) helps to identify high-risk pregnant women, optimise GDM risk stratification, and develop targeted prevention strategies. However, the single-center design and exclusive focus on Korean women limit generalizability. Future studies need validation in diverse populations, as well as dynamic monitoring and specifically designed prospective research to strengthen the evidence.

## Data Availability

Details of the publicly available datasets analyzed in this study are included in the article/Supplementary material. Further inquiries can be directed to the corresponding author.

## References

[1] Modzelewski R Stefanowicz-Rutkowska MM Matuszewski W Bandurska-Stankiewicz EM. Gestational diabetes mellitus-recent literature review. J Clin Med. (2022) 11:5736. doi: 10.3390/jcm11195736, 36233604 PMC9572242

[2] Kim K-S Hong S Han K Park C-Y. The clinical characteristics of gestational diabetes mellitus in Korea: a National Health Information Database Study. Endocrinol Metab Seoul Korea. (2021) 36:628–36. doi: 10.3803/EnM.2020.948, 34034366 PMC8258326

[3] Seneviratne SN Rajindrajith S. Fetal programming of obesity and type 2 diabetes. World J Diabetes. (2022) 13:482–97. doi: 10.4239/wjd.v13.i7.482, 36051425 PMC9329845

[4] Harnois-Leblanc S Hivert M-F. Stopping the intergenerational risk of diabetes-from mechanisms to interventions: a report on research supported by pathway to stop diabetes. Diabetes. (2025) 74:255–64. doi: 10.2337/dbi24-0014, 39556447

[5] Guglielmini G Falcinelli E Piselli E Mezzasoma AM Tondi F Alfonsi L . Gestational diabetes mellitus is associated with in vivo platelet activation and platelet hyperreactivity. Am J Obstet Gynecol. (2025) 232:120.e1–120.e14. doi: 10.1016/j.ajog.2024.04.003, 38582292

[6] Wicklow B Retnakaran R. Gestational diabetes mellitus and its implications across the life span. Diabetes Metab J. (2023) 47:333–44. doi: 10.4093/dmj.2022.0348, 36750271 PMC10244196

[7] Mao Y Hu W Xia B Liu L Han X Liu Q. Association between gestational diabetes mellitus and the risks of type-specific cardiovascular diseases. Front Public Health. (2022) 10:940335. doi: 10.3389/fpubh.2022.940335, 35865249 PMC9294140

[8] Metzger BE Gabbe SG Persson B Buchanan TA Catalano PA Damm P . International association of diabetes and pregnancy study groups recommendations on the diagnosis and classification of hyperglycemia in pregnancy. Diabetes Care. (2010) 33:676–82. doi: 10.2337/dc09-1848 20190296 PMC2827530

[9] D’Arcy RJ Cooke IE McKinley M McCance DR Graham UM. First-trimester glycaemic markers as predictors of gestational diabetes and its associated adverse outcomes: a prospective cohort study. Diabet Med J Br Diabet Assoc. (2023) 40:e15019. doi: 10.1111/dme.15019, 36453695 PMC10107539

[10] Mou SS Gillies C Hu J Danielli M Al Wattar BH Khunti K . Association between HbA1c levels and fetal macrosomia and large for gestational age babies in women with gestational diabetes mellitus: a systematic review and Meta-analysis of 17,711 women. J Clin Med. (2023) 12:3852. doi: 10.3390/jcm12113852, 37298047 PMC10253627

[11] Zhang J Suo Y Wang L Liu D Jia Y Fu Y . Association between atherogenic index of plasma and gestational diabetes mellitus: a prospective cohort study based on the Korean population. Cardiovasc Diabetol. (2024) 23:237. doi: 10.1186/s12933-024-02341-9, 38970008 PMC11227226

[12] Venkatesh KK Perak AM Wu J Catalano P Josefon JL Costantine MM . Impact of hypertensive disorders of pregnancy and gestational diabetes mellitus on offspring cardiovascular health in early adolescence. Am J Obstet Gynecol. (2025) 232:218.e1–218.e12. doi: 10.1016/j.ajog.2024.04.037, 38703941

[13] Yang M-N Zhang L Wang W-J Huang R He H Zheng T . Prediction of gestational diabetes mellitus by multiple biomarkers at early gestation. BMC Pregnancy Childbirth. (2024) 24:601. doi: 10.1186/s12884-024-06651-4, 39285345 PMC11406857

[14] Razo-Azamar M Nambo-Venegas R Meraz-Cruz N Guevara-Cruz M Ibarra-Gonzalez I Vela-Amieva M . An early prediction model for gestational diabetes mellitus based on metabolomic biomarkers. Diabetol Metab Syndr. (2023) 15:116. doi: 10.1186/s13098-023-01098-7, 37264408 PMC10234027

[15] Xie Y-P Lin S Xie B-Y Zhao H-F. Recent progress in metabolic reprogramming in gestational diabetes mellitus: a review. Front Endocrinol. (2023) 14:1284160. doi: 10.3389/fendo.2023.1284160, 38234430 PMC10791831

[16] Martin-Saladich Q Simo R Ciudin A Baguna J Ramirez-Serra C Aguade-Bruix S . Phenotypic patterns of metabolic dysfunction-associated steatotic liver disease in type 2 diabetes: the impact of insulin. Eur J Clin Investig. (2025) 55:e70050. doi: 10.1111/eci.70050, 40251759 PMC12362049

[17] Barrera F Uribe J Olvares N Huerta P Cabrera D Romero-Gomez M. The Janus of a disease: diabetes and metabolic dysfunction-associated fatty liver disease. Ann Hepatol. (2024) 29:101501. doi: 10.1016/j.aohep.2024.101501, 38631419

[18] Lee K-C Wu P-S Lin H-C. Pathogenesis and treatment of non-alcoholic steatohepatitis and its fibrosis. Clin Mol Hepatol. (2023) 29:77–98. doi: 10.3350/cmh.2022.0237, 36226471 PMC9845678

[19] Yang Z Li A Jiang Y Maidaiti X Wu Y Jin Y. Global burden of metabolic dysfunction-associated steatotic liver disease attributable to high fasting plasma glucose in 204 countries and territories from 1990 to 2021. Sci Rep. (2024) 14:22232. doi: 10.1038/s41598-024-72795-0, 39333707 PMC11437073

[20] Sabatini S Sen P Carli F Pezzica S Rosso C Lembo E . Hepatic glucose production rises with the histological severity of metabolic dysfunction-associated steatohepatitis. Cell Rep Med. (2024) 5:101820. doi: 10.1016/j.xcrm.2024.101820, 39566466 PMC11604487

[21] Zhang L Gao S Luan Y Su S Zhang E Liu J . Predictivity of hepatic steatosis index for gestational hypertension and preeclampsia: a prospective cohort study. Int J Med Sci. (2025) 22:834–44. doi: 10.7150/ijms.104943, 39991765 PMC11843148

[22] Si R Xiao J Zheng K Yin Y Li Y. Association between the hepatic steatosis index and risk of incident type 2 diabetes mellitus in the normoglycemic population: a longitudinal prospective study in Japan. Diabetes Metab Syndr Obes Targets Ther. (2024) 17:2317–26. doi: 10.2147/DMSO.S462459, 38863519 PMC11166155

[23] Chen K Pan Y Xiang X Meng X Yao D Lin L . The nonalcoholic fatty liver risk in prediction of unfavorable outcome after stroke: a nationwide registry analysis. Comput Biol Med. (2023) 157:106692. doi: 10.1016/j.compbiomed.2023.106692, 36924734

[24] Zhang S Wang L Yu M Guan W Yuan J. Fat mass index as a screening tool for the assessment of non-alcoholic fatty liver disease. Sci Rep. (2022) 12:20219. doi: 10.1038/s41598-022-23729-1, 36418352 PMC9684573

[25] Zhu Y Hu H Wu Y Rao Y Li Q Duan X . The association between fatty liver index and onset of diabetes: secondary analysis of a population-based cohort study. BMC Public Health. (2023) 23:679. doi: 10.1186/s12889-023-15442-z, 37041534 PMC10091632

[26] Fan J-G Kim S-U Wong VW-S. New trends on obesity and NAFLD in Asia. J Hepatol. (2017) 67:862–73. doi: 10.1016/j.jhep.2017.06.003 28642059

[27] Rinella ME Lazarus JV Ratziu V Francque SM Sanyal AJ Kanwal F . A multisociety Delphi consensus statement on new fatty liver disease nomenclature. J Hepatol. (2023) 79:1542–56. doi: 10.1016/j.jhep.2023.06.003, 37364790

[28] Lee SM Kim BJ Koo JN Norwitz ER Oh IH Kim SM . Nonalcoholic fatty liver disease is a risk factor for large-for-gestational-age birthweight. PLoS One. (2019) 14:e0221400. doi: 10.1371/journal.pone.0221400, 31449538 PMC6709883

[29] Gastaldelli A Kozakova M Højlund K Flyvbjerg A Favuzzi A Mitrakou A . Fatty liver is associated with insulin resistance, risk of coronary heart disease, and early atherosclerosis in a large European population. Hepatol Baltim Md. (2009) 49:1537–44. doi: 10.1002/hep.22845, 19291789

[30] Lee J-H Kim D Kim HJ Lee C-H Yang JI Kim W . Hepatic steatosis index: a simple screening tool reflecting nonalcoholic fatty liver disease. Dig Liver Dis. (2010) 42:503–8. doi: 10.1016/j.dld.2009.08.002 19766548

[31] American College of Obstetricians and Gynecologists. ACOG practice bulletin no. 190: gestational diabetes mellitus. Obstet Gynecol. (2018) 131:e49–64. doi: 10.1097/AOG.0000000000002501 29370047

[32] Wallace TM Levy JC Matthews DR. Use and abuse of HOMA modeling. Diabetes Care. (2004) 27:1487–95. doi: 10.2337/diacare.27.6.1487, 15161807

[33] Cai X Gao J Liu S Wang M Hu J Hong J . Hepatic steatosis index and the risk of type 2 diabetes mellitus in China: insights from a general population-based cohort study. Dis Markers. (2022) 2022:1–10. doi: 10.1155/2022/3150380, 35968500 PMC9365599

[34] Linder T Eppel D Kotzaeridi G Rosicky I Yerlikaya-Schatten G Kiss H . Fatty liver indices and their association with glucose metabolism in pregnancy - an observational cohort study. Diabetes Res Clin Pract. (2022) 189:109942. doi: 10.1016/j.diabres.2022.109942, 35691476

[35] Lee SM Kwak SH Koo JN Oh IH Kwon JE Kim BJ . Non-alcoholic fatty liver disease in the first trimester and subsequent development of gestational diabetes mellitus. Diabetologia. (2019) 62:238–48. doi: 10.1007/s00125-018-4779-8, 30470912

[36] Castera L Friedrich-Rust M Loomba R. Noninvasive assessment of liver disease in patients with nonalcoholic fatty liver disease. Gastroenterology. (2019) 156:1264–1281.e4. doi: 10.1053/j.gastro.2018.12.036, 30660725 PMC7505052

[37] Jeong S Park SJ Na SK Park SM Song B-C Oh YH. Validity of fatty liver prediction scores for diagnosis of fatty liver by Fibroscan. Hepatobiliary Pancreat Dis Int HBPD INT. (2024) 23:353–60. doi: 10.1016/j.hbpd.2023.02.009, 36870896

[38] Samuel VT Shulman GI. Mechanisms for insulin resistance: common threads and missing links. Cell. (2012) 148:852–71. doi: 10.1016/j.cell.2012.02.017, 22385956 PMC3294420

[39] Abulizi A Camporez J-P Zhang D Samuel VT Shulman GI Vatner DF. Ectopic lipid deposition mediates insulin resistance in adipose specific 11β-hydroxysteroid dehydrogenase type 1 transgenic mice. Metabolism. (2019) 93:1–9. doi: 10.1016/j.metabol.2018.12.003, 30576689 PMC6401251

[40] Powe CE Huston Presley LP Locascio JJ Catalano PM. Augmented insulin secretory response in early pregnancy. Diabetologia. (2019) 62:1445–52. doi: 10.1007/s00125-019-4881-6, 31177313 PMC6786902

[41] Hivert M-F Backman H Benhalima K Catalano P Desoye G Immanuel J . Pathophysiology from preconception, during pregnancy, and beyond. Lancet. (2024) 404:158–74. doi: 10.1016/S0140-6736(24)00827-4, 38909619

[42] Zhang Z Zhou Z Li H. The role of lipid dysregulation in gestational diabetes mellitus: early prediction and postpartum prognosis. J Diabetes Investig. (2024) 15:15–25. doi: 10.1111/jdi.14119, 38095269 PMC10759727

[43] Wang G Buckley JP Bartell TR Hong X Pearson C Wang X. Gestational diabetes mellitus, postpartum Lipidomic signatures, and subsequent risk of type 2 diabetes: a Lipidome-wide association study. Diabetes Care. (2023) 46:1223–30. doi: 10.2337/dc22-1841, 37043831 PMC10234741

[44] Ciardullo S Bianconi E Zerbini F Perseghin G. Current type 2 diabetes, rather than previous gestational diabetes, is associated with liver disease in U.S. women. Diabetes Res Clin Pract. (2021) 177:108879. doi: 10.1016/j.diabres.2021.108879, 34058299

[45] Kvist AAS Sharma A Sommer C Qvigstad E Gulseth HL Sollid ST . Adipose tissue insulin resistance in south Asian and Nordic women after gestational diabetes mellitus. Meta. (2024) 14:288. doi: 10.3390/metabo14050288, 38786765 PMC11123011

[46] Piechota W Staszewski A. Reference ranges of lipids and apolipoproteins in pregnancy. Eur J Obstet Gynecol Reprod Biol. (1992) 45:27–35. doi: 10.1016/0028-2243(92)90190-a, 1618359

