# Supplementary material for: First-trimester fatty liver index and hepatic steatosis index independently predict gestational diabetes risk: a prospective cohort study
Source: Front Nutr. 2026 Mar 19;13:1769688. doi: 10.3389/fnut.2026.1769688 (PMC13045505; doi:10.3389/fnut.2026.1769688)
Supplement: Supplementary file 1 [file Table_1.docx]

Table S1 Supplementary indicator of FLI and HSI

| Variable | Nagelkerke R² | Hosmer–Lemeshow Test | Brier Score |
| --- | --- | --- | --- |
| FLI | 0.105 | χ² = 8.348, P = 0.400 | 0.048 |
| HSI | 0.092 | χ² = 6.458, P = 0.596 | 0.051 |
