# Supplementary material for: First-trimester fatty liver index and hepatic steatosis index independently predict gestational diabetes risk: a prospective cohort study
Source: Front Nutr. 2026 Mar 19;13:1769688. doi: 10.3389/fnut.2026.1769688 (PMC13045505; doi:10.3389/fnut.2026.1769688)
Supplement: Supplementary file 2 [file Table_2.docx]

Table S2 Cross-Validation and Optimal Cut-off values of FLI and HSI

| Indicator | FLI | HSI |
| --- | --- | --- |
| Sensitivity (95% CI) | 80.56% (67.8%, 93.4%) | 83.33% (68.6%, 93.0%) |
| Specificity (95% CI) | 80.45% (77.1%, 83.9%) | 65.36% (61.2%, 69.3%) |
| Cross-Validation Mean C-index | 0.813 | 0.787 |
| Cross-Validation Mean Brier Score | 0.052 | 0.053 |
| Cutoff Value | 23.33 | 31.13 |
